# Targeted Genotyping of MIS-C Patients Reveals a Potential Alternative Pathway Mediated Complement Dysregulation during COVID-19 Infection

**DOI:** 10.3390/cimb44070193

**Published:** 2022-06-28

**Authors:** Eleni Gavriilaki, Stefanos A. Tsiftsoglou, Tasoula Touloumenidou, Evangelia Farmaki, Paraskevi Panagopoulou, Elissavet Michailidou, Evaggelia-Evdoxia Koravou, Ioulia Mavrikou, Elias Iosifidis, Olga Tsiatsiou, Eleni Papadimitriou, Efimia Papadopoulou-Alataki, Penelope Georgia Papayanni, Christos Varelas, Styliani Kokkoris, Apostolia Papalexandri, Maria Fotoulaki, Assimina Galli-Tsinopoulou, Dimitrios Zafeiriou, Emmanuel Roilides, Ioanna Sakellari, Achilles Anagnostopoulos, Athanasios Tragiannidis

**Affiliations:** 1 Hematology Department & BMT Unit, G Papanicolaou Hospital, 57010 Thessaloniki, Greece; tasoula.touloumenidou@gmail.com (T.T.); evakikor@gmail.com (E.-E.K.); iouliamavrikou@gmail.com (I.M.); papagianni.pinelopi@gmail.com (P.G.P.); varelaschris@gmail.com (C.V.); lila.papalexandri@gmail.com (A.P.); bmt@gpapanikolaou.gr (I.S.); achanag@gmail.com (A.A.); 2 Laboratory of Pharmacology, Department of Pharmacy, Aristotle University of Thessaloniki, 54124 Thessaloniki, Greece; 3 1st Pediatric Department, Aristotle University of Thessaloniki, Hipporkation Hospital, 54642 Thessaloniki, Greece; farmaki@auth.gr (E.F.); eleni.papadimitriou@gmail.com (E.P.); dizafeir@auth.gr (D.Z.); 4 4th Pediatric Department, Aristotle University of Thessaloniki, Papageorgiou Hospital, 56429 Thessaloniki, Greece; ppanagopoulou@auth.gr (P.P.); papadoef@auth.gr (E.P.-A.); mfotoula@auth.gr (M.F.); 5 3rd Pediatric Department, Aristotle University of Thessaloniki, Hippokration Hospital, 54642 Thessaloniki, Greece; elisavet.michailidou@gmail.com (E.M.); iosifidish@gmail.com (E.I.); tsiatsiouo@gmail.com (O.T.); roilides@auth.gr (E.R.); 6 Laboratory of Hematology and Hospital—Blood Transfusion Unit, Medical School, University General Hospital “Attikon”, NKUA, 12462 Athens, Greece; stellakok@gmail.com; 7 2nd Pediatric Department, Aristotle University of Thessaloniki, AHEPA Hospital, 54621 Thessaloniki, Greece; agalli@auth.gr (A.G.-T.); atragian@auth.gr (A.T.)

**Keywords:** COVID-19, MIS-C, children, complement, SNPs

## Abstract

Complement dysregulation has been documented in adults with COVID-19 and implicated in relevant pediatric inflammatory responses against SARS-CoV-2. We propose that signatures of complement missense coding SNPs associated with dysregulation could also be identified in children with multisystem inflammatory syndrome (MIS-C). We investigated 71 pediatric patients with RT-PCR validated SARS-CoV-2 hospitalized in pediatric COVID-19 care units (November 2020–March 2021) in three major groups. Seven (7) patients suffered from MIS-C (MIS-C group), 32 suffered from COVID-19 and were hospitalized (admitted group), whereas 32 suffered from COVID-19, but were sent home. All patients survived and were genotyped for variations in the *C3*, *C5*, *CFB*, *CFD*, *CFH*, *CFHR1*, *CFI*, *CD46*, *CD55*, *MASP1*, *MASP2*, *MBL2*, *COLEC11*, *FCN1*, and *FCN3* genes. Upon evaluation of the missense coding SNP distribution patterns along the three study groups, we noticed similarities, but also considerably increased frequencies of the alternative pathway (AP) associated with SNPs rs12614 *CFB*, rs1061170, and rs1065489 *CFH* in the MIS-C patients. Our analysis suggests that the corresponding substitutions potentially reduce the C3b-inactivation efficiency and promote slower and weaker AP C3bBb pre-convertase assembly on virions. Under these circumstances, the complement AP opsonization capacity may be impaired, leading to compromised immune clearance and systemic inflammation in the MIS-C syndrome.

## 1. Introduction

Since the beginning of the COVID-19 (Coronavirus disease 2019) outbreak, a large number of studies have attempted to dissect the complex molecular and cellular basis of the SARS-CoV-2 (severe acute respiratory syndrome coronavirus 2) induced pathophysiology [1]. As aspects of the COVID-19 pathology resemble complementopathies [2,3,4,5] and complement-mediated thrombotic microangiopathy [6,7], it became apparent relatively early that complement may influence viral sensing and inflammation dynamics during infection [8].

**Figure 1:**
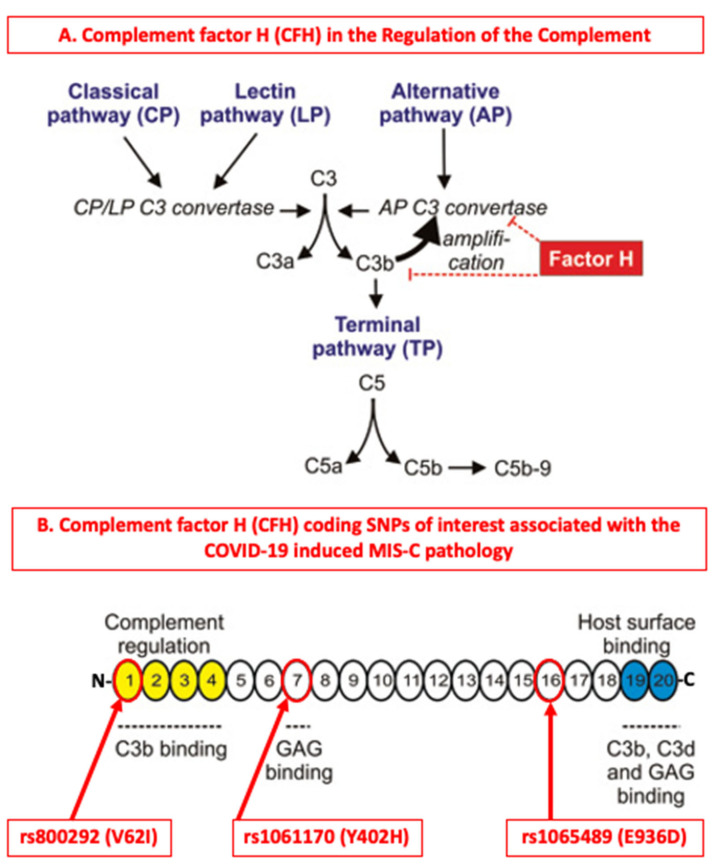
The contributions of complement factor H (CFH) and its associated coding SNPs in the regulation of the complement system. (**A**) Complement factor H (CFH) is one of the major regulators of the complement system by exhibiting decay accelerating activity against the C3bBb alternative pathway (AP) convertase and by acting as the major soluble cofactor for the complement factor I (CFI) mediated fragmentation of C3(H_2_O)/C3b [37]. The cleavage products iC3b, C3dg, and C3d are major opsonins of the complement system [38]. (**B**) Domain topography of the human CFH and the highlighting of coding SNPs of interest associated with the MIS-C pathology. Each numbered circle corresponds to one of the 20 complement control protein (CCP) (or short consensus repeat (SCR) or Sushi) domains of CFH. N- for the -NH_2_ terminal end, -C for the -COOH terminal end. GAGs: Glucosamino glycans, MIS-C: Multisystem Inflammatory Syndrome in Children. This image originates from the Fülöp, TG et al. 2015 with modifications [39].

Targeting complement in COVID-19 has been under discussion more extensively [9,10,11] as several elegant studies have shown that SARS-CoV-2 can directly activate complement by engaging its multiple pattern recognition components [12,13,14,15,16,17,18,19]. Experimental studies of past coronavirus infections have shown that such viruses (SARS-CoV, MERS-CoV and SARS-CoV-2) can bind mannose-binding protein-associated serine protease 2 (MASP-2) and induce complement mediated inflammatory lung damage [20], while the inhibition of complement C3 can significantly reduce the inflammatory responses to pulmonary infections [21,22]. The activation of the alternative pathway by the spike glycoprotein S of SARS-CoV-2 has been of particular interest as the spike promotes entry of the virus into cells and is a major antigenic target for B cell responses [14,23,24,25]. Furthermore, the potential exploitation of the activation of the alternative pathway via the amplification loop by SARS-CoV-2 is of even greater interest [26] (Figure 1), because it can explain to a considerable extent the gradual diminishment of complement responses during the systemic establishment of the virus, especially in severe cases of infection [5,27,28].

In younger ages, the majority of symptomatic COVID-19 pediatric patients exhibit low fever and mild symptoms that usually involve the upper and sometimes the lower respiratory system. Similarly to adults, some children may develop a severe infection characterized by respiratory failure, myocarditis, renal involvement, shock, and diabetic ketoacidosis as well as severe hematologic and gastrointestinal disorders [6,29,30,31,32,33]. Numerous studies have demonstrated the role of complement activation in the pathophysiology of various childhood diseases such as the atypical uremic hemolytic syndrome, the catastrophic antiphospholipid syndrome, thrombotic microangiopathy after transplantation, and the Hemolysis, Elevated Liver enzymes and Low Platelets (HELLP) syndrome [2,34]. A common feature of these disorders is the genetic predisposition to complement activation, which is the source of their pathophysiology [2]. The onset of the syndrome requires the triggering of complement activation (pregnancy, inflammation, surgery, autoimmunity), which acts as the initiator of pathophysiology. The main injuries mediated by complement in thrombotic microangiopathy are endothelial dysfunction and small vessel thrombosis. Complement also interacts with the inflammatory response and neutrophil activation, which may also occur in severe COVID-19 infection, both in the heart [35] and in the kidneys [4].

In line with the data on adults, a few studies have also supported the involvement of complement deregulation in severe COVID-19 in pediatric patients, which primarily manifests as SARS-CoV-2 induced Multisystem Inflammatory Syndrome in Children (MIS-C) (CDC, HAN Archive-00432) [36]. Our central aim was to identify complement coding SNPs of interest in patients with COVID-19 induced MIS-C or Kawasaki-like MIS-C pathologies (CDC, HAN Archive-00432) and compare their distributions in pediatric patients with acute COVID-19 that required admission or not.

## 2. Materials and Methods

### 2.1. Study Population

Our study population consisted of consecutive Caucasian newborns, infants, children, and adolescents <18 years, all diagnosed with COVID-19 infection in four different pediatric clinics in Thessaloniki. We did not include any matched healthy and non-infected individuals in our study. The demographic data, comorbidities, clinical and laboratory findings (WBC, Hb, Ht, PLTs, fibrinogen, d-dimers, CRP, erythrocyte sedimentation rate/ESR, procalcitonin, and liver and kidney function) were recorded for each patient. The study was conducted according to the guidelines of the Declaration of Helsinki, and approved by the Institutional Review Board of Aristotle University of Thessaloniki. Informed consent was obtained from all subjects involved in the study. According to the CDC criteria (HAN Archive-00432), COVID-19 induced MIS-C was defined as follows. (1) Patients <21 years of age with fever >38.0 °C for ≥24 h, laboratory findings of inflammation such as increased CRP, erythrocyte sedimentation rate, fibrinogen, d-dimer, ferritin, LDH, IL-6 and/or WBC as well as a decrease in lymphocytes and serum albumin. In addition, patients developed symptoms that required hospitalization with a concomitant multisystem involvement (>2) such as heart, kidney, lung, hematopoietic, gastrointestinal, skin, or central nervous system/CNS). (2) Absence of another possible diagnosis. (3) Positive or recently positive SARS-CoV-2 infection by RT-PCR or exposure to a suspected or confirmed case within four weeks before the onset of symptoms. Finally, some patients met all or part of the Kawasaki disease criteria [40].

### 2.2. Genomic Analysis

Genomic DNA was isolated from peripheral blood using the QIAamp DNA Mini Blood (Qiagen, Hilden, Germany). This was analyzed using Next Generation Sequencing (NGS) specific for the 15 complement genes *C3*, *C5*, *CFB*, *CFD*, *CFH*, *CFHR1*, *CFI*, *CD46*, *CD55*, *MASP1, MASP2, MBL2, COLEC11, FCN1*, and *FCN3*, as previously published in two studies of adult patients by the team at the Hematology Clinic of Papanikolaou Hospital [41]. The probes and primers were designed in DesignStudio (Illumina, San Diego, CA, USA) to cover all exonic regions spanning 15 bases into introns (98% coverage). A total of 10 ng of genomic DNA was used to build the libraries (MiniSeq, Illumina). Libraries were quantified using Qubit (Thermo Fisher Scientific, Waltham, MA, USA) and sequenced on a MiniSeq System in a 2 × 150 bp run (Illumina). Sequence quality was initially assessed using Illumina tools. As the original sequencing was of high quality, each sample was processed independently, in order to properly map the sequences against the human reference genome. Variations with an allelic frequency higher than 20% were analyzed. Both Ensembl and Refseq resources were used for annotation of the output files.

### 2.3. Data Mining and Clustering

We carried out two independent sets of refinement analysis for the coding variants, one for the nine genes associated with the activation and regulation of the alternative pathway (*C3, C5, CFB, CFD, CFH, CFHR1, CFI, CD46, CD55*), and one for the six genes associated with the activation of the lectin pathway (*MASP1, MASP2, MBL2, COLEC11, FCN1* and *FCN3*). We first identified all of the detected missense coding SNPs of the genes from each annotated output patient genotyping profile and evaluated them with publicly available databases (NCBI ClinVar, dbSNP, and UniProt). For each analysis set, the sum of the identified coding SNPs was then clustered in three major groups based on the clinical outcome of the infection. One major group included the coding SNPs from patients who were not-admitted (*nN* = 32), one from patients who were hospitalized (*nY* = 32), and another from patients who were hospitalized and exhibited COVID-19 induced MIS-C pathologies (the MIS-C group) (*nM* = 7). Within this frame, the sum of the coding SNPs identified in our study was segregated using as the sole criterium the terminal clinical outcome of infection based on the CDC guidelines describing the COVID-19 induced MIS-C pathologies (HAN Archive-00432).

For every coding SNP identified, its relative variant frequency (%) was calculated by dividing the number of times the coding SNP was represented within each group, with the total number of patients included in the group. For the sum of the coding SNPs identified, analysis effort was put toward identifying common groups of SNPs as well as unique SNPs for each group. With the term common SNPs, we refer to all of the coding SNPs identified across all three groups of our study, either for the alternative pathway or the lectin pathway genes. With the term unique SNPs, we refer to all coding SNPs that were identified specifically in each of the three study groups. The results for each pathway genes are listed separately in two independent tables.

### 2.4. Statistical Analysis

The final genetic analysis file was used to statistically evaluate the variants detected by the Statistical Package for Social Sciences (SPSS), version 22. For each analysis set, the sum of the identified coding SNPs was then clustered into three major groups based on the clinical outcome of the infection. One major group included the coding SNPs from patients who had acute COVID-19 but were non-admitted, one from patients who also had acute COVID-19 and were admitted, and another from patients who were hospitalized due to COVID-19 induced MIS-C (MIS-C group). The following clinical parameters were taken into account: gender, age, presence of fever, dyspnea, cough or other pulmonary difficulties and symptoms, routine laboratory tests, treatment, and hospitalization. The frequency of each genomic variation was calculated and the results presented by descriptive statistical methods. The chi-square test was applied to compare the qualitative variables, while the Student’s *t*-test for the normal or the Mann–Whitney U test for the non-normal distribution were applied to compare the quantitative ones. Statistical tests were 2-sided with *p*-values ≤ 0.05 denoting statistical significance. For the sake of simplicity, only statistically significant *p*-values were included in the Results section.

## 3. Results

### 3.1. Study Population and Identification of Coding SNPs of Clinical Significance

A total of 71 patients (*nT* = 71) with a median age of 7 years (range 0.3–17 years) were studied, seven of which developed COVID-19 induced multisystemic inflammatory syndrome (MIS-C and Kawasaki-like MIS-C) according to the above criteria of the CDC. Of the remaining 64, 32 required hospitalization, while all patients survived. Table 1 summarizes the major patient characteristics of our study. As our study did not include any matched healthy and non-infected individuals, all comparisons and conclusions were based on intra analyses between the tree major study groups. It should also be noted that the sub-groups of patients, especially the MIS-C group, were rather small, limiting the generalization of results in larger populations. 

In our preliminary analysis of the whole population under study, variants of various clinical significance were identified among the extended diversity of the SNPs that we managed to detect: pathological or potentially pathological, benign or potentially benign, and of uncertain clinical significance (median: 97 variants, range 61–103). Among the coding SNPs of potential clinical significance that have been described in endothelial dysfunction syndromes, we identified the risk factor variant, rs2230199 of C3 in 28 patients and the risk factor variant rs800292 of CFH in 36 patients out of a total of 71 individuals (ClinVar) (Table 2). In addition, 22 patients exhibited the pathogenetic coding SNP rs1800450 of MBL2 and nine showed the pathogenetic deletion rs532781899 of FCN3, previously associated with inflammatory syndromes (ClinVar) (Table 3). The sum of our analysis for missense coding SNPs among all patients revealed eight common and 18 unique alternative pathway associated SNPs (Table 2) as well as nine common and eight unique lectin pathway associated SNPs (Table 3). Interestingly, among the alternative pathway associated coding SNPs, we did not identify any common ones for CFD, CD46, and CD55, while among the lectin pathway associated SNPs, none were common for FCN1. The vast majority of all the unique coding SNPs identified were sporadic in terms of abundance, but some might potentially contribute to the dynamics of complement regulation in some individuals.

When we further analyzed the frequencies of the missense coding SNPs identified for all three major groups of our study, we recorded SNPs present in relatively high frequencies (>70%) in all three major groups (rs11098044 of CFI, rs1061170 of CFH, rs12711521 of MASP2) (Table 2 and Table 3) as well as other less common, but with distinctly different frequencies among the patients who needed hospitalization and developed inflammatory syndromes.

### 3.2. Common Coding SNPs with Similar and Different Frequencies

**Figure 2:**
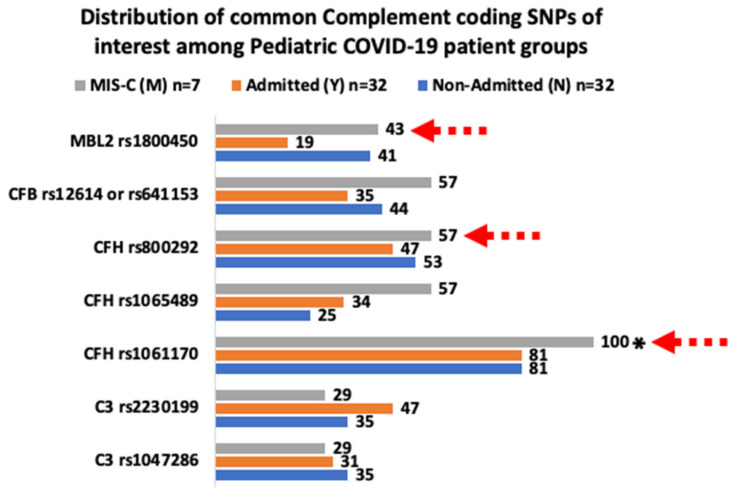
The graph visualizes the data derived from Table 2 and Table 3. The red dashed arrows indicate the most striking differences observed for the frequencies of CFB rs12614, rs641153, and CFH rs1061170, rs1065489 among the pediatric COVID-19 patient groups examined. * p = 0.007 for the comparison between the MIS-C and non-MIS-C patients as shown in Table 2.

In order to interpret the frequency patterns observed between the two common classes of coding SNPs (AP and LP common SNPs) in our three study groups, we attempted pairwise comparisons of the groups for each class. Upon the evaluation of the distribution patterns along our three study groups, we noticed considerably increased frequencies of the alternative pathway (AP) associated SNPs rs12614 of CFB, the rs1061170 and rs1065489 of CFH in the MIS-C patients as well as of the rs2230199 of C3 in the admitted patients (Table 2, Figure 2). Compared to the non-admitted (N) and the MIS-C (M), the admitted patients (Y) also exhibited a decreased frequency, nearly halved, of the rs1800450 MBL2 lectin pathway (LP) coding SNP (Table 3, Figure 2). Compared to the non-admitted (N) group, the combined increased frequencies of rs12614 of CFB, rs1061170, and rs1065489 of CFH in the MIS-C group appeared interesting, as the frequencies of the rs1047286 and rs2230199 of C3 appeared similar between the two groups (Table 2). The frequency of the SNP rs12614 of CFB was the highest in the MIS-C group (57%) (*nM* = 7) compared to the sum of the rs12614 and rs641153 of CFB in the other two groups (each *n* = 32) (Table 2). The SNP rs1061170 was detected in all of the examined MIS-C patients (100%, *p* = 0.007), while the SNP rs1065489 of CFH was present in the MIS-C group in a nearly double (~2X) frequency (57%) compared to the other two groups, 25% for the N and 34% for the Y group, respectively (Table 2). We also noticed that in 3 out of the 4 MIS-C patients with rs12614 of CFB, the rs12614 was co-present along the rs1061170, rs1065489 and rs800292 of CFH (See Supplementary Materials). For plain comparison purposes, as a value, the added frequency percentages of the rs1061170, rs1065489 and rs800292 of the CFH coding SNPs among the three groups, was the highest in MIS-C patients (214%) (*nM* = 7) and considerably lower and similar among the N (159%) (*nN* = 32) and Y (162%) (*nY* = 32) groups.

Among the common LP variants, the MIS-C group appeared to lack a few of the common variants that appeared in the N and Y groups, but the size of this group (*nM* = 7) is relatively small for the generalization of all or some of these absences (Table 3). This was similar for the increased presence of the rs7567833 of COLEC11 in the MIS-C group that was naturally low in abundance in the non-admitted (N) group. Compared to the other two groups, the reduced presence of the rs1800450 of MBL2 in the admitted group (Y) could be indicative of a more active and potent lectin pathway in some individuals of this group (Table 3). The rs1800450 corresponding amino acid substitution (G54D) has been associated with low concentrations of functional serum mannose-binding protein (MBP) and increased susceptibility to recurrent infections including viral persistence in the pathogenesis of acute hepatitis B (UniProt: P11226 polymorphisms) [42].

## 4. Discussion

The higher susceptibility to develop MIS-C in older children, adolescents, and young adults may be due to the maturity of the immune system, which may be more able to mount a hyperinflammatory response in comparison to younger children and older adults. Another possible factor for the lower incidence of MIS-C in younger children could be the lower expression of the cell surface enzyme angiotensin-converting enzyme 2 (ACE2) [43].

As our pilot study did not include any matched healthy and non-infected individuals, all comparisons and conclusions were based on intra analyses between the three major study groups. Among the coding SNPs identified as important for complement regulation in the MIS-C group were rs1061170, rs1065489, rs800292, corresponding to complement factor H (CFH), which has multidimensional roles [37] (Figure 1). CFH binds to antigenic surfaces by sialic acid-containing glucosamino glycans (GAGs) [44] or by binding to the surface-attached CRP, thereby increasing the affinity of CFH for C3b [45]. The increased affinity enhances the complement regulation through the complement factor I (CFI) mediated downregulation of the C3bBb AP convertase and the generation of iC3b, C3dg, and C3d, which are major opsonins of the complement system [38].

The rs1061170 coding SNP (Y402H in CCP7) has been linked with increased disease risk for age-related macular degeneration (AMD) [46,47,48] and was found to be highly abundant (100%) in all of our examined MIS-C patients (*nM* = 7) (Table 2, 100%, *p* = 0.007). It is structurally associated with the reduced binding affinity of the Y402H variant with multiple complement activators, regulators, and ligands including heparin [44] and the C-reactive protein (CRP) [45] (Figure 1). Therefore, in terms of viral clearance, the reduced binding affinity of the Y402H variant could be associated with a decreased effectiveness of CFH in the downregulation of the C3bBb AP convertase and weaker opsonization dynamics during infection. Although this pattern is interesting, the small size of the MIS-C group (*nM* = 7) does not allow for broader generalization of this observation.

The rs1065489 (E936D) coding SNP is located in the gene region encoding the CCP16 domain (Figure 1). Removal of the C-terminal CCP domains 16–20 showed that it dramatically localized the binding of CFH to non-activating surfaces by 90%, highlighting its importance for host recognition [49]. Although the CCP16 domain has not yet been found to directly harbor any distinct ligand binding sites [37], it is neighbors with a recently characterized dimerization site at CCP17 and CCP18 that is implicated in the oligomerization and self-assembly of CFH upon its binding to surfaces through the C-terminal CCP19 and 20 domains [50]. These terminal domains support the binding of sialic acids and heparin [44]. The SARS-CoV-2 S gene encodes 22 N-linked glycan sequons per protomer, which is likely to play a role in protein folding and immune evasion [25]. Therefore, similarly to rs1061170, the reduced binding affinity of the rs1065489 (E936D) SNP could also contribute to the decreased contribution dynamics of CFH in the downregulation of the C3bBb AP convertase and the opsonization processes during infection. In our study, among the three groups studied, the frequency of rs1065489 was the highest in the MIS-C group (57%) (*nM* = 7) (Table 2).

The rs800292 (V62I in CCP1) coding SNP is a gain of function variant as in vitro experiments have shown that the protein resulting from the minor allele A I62 binds more efficiently to the fluid phase and surface-bound C3b than the protein resulting from the G allele V62, and also competes better with factor B in the formation of proconvertase [51]. Therefore, the I62 variant can decrease the proconvertase formation dynamics and catalyze the inactivation of the fluid-phase and surface-bound C3b more efficiently than the V62 variant [51] (Figure 1). Therefore, the rs800292 (V62I in CCP1) SNP is a potentially important influencer of the C3-CFB proconvertase association dynamics and its enhanced abundance in individuals can be protective against frequent or background activation stimuli of the alternative pathway (AP). In our study, among the three groups studied, the frequency of rs800292 was also the highest in the MIS-C group (57%) (*nM* = 7) (Table 2).

Overall, based on the evidence presented and discussed, the increased presence of the rs1061170, rs1065489 and rs800292 CFH coding SNPs in the MIS-C group synergistically suggests a reduced capacity of CFH in the downregulation of the C3bBb AP convertase and the opsonization processes during infection (Figure 1). However, for the case of the MIS-C group, the increased abundance of the rs12614/rs641153 R32 coding SNPs (57%), compared to the other two groups (Table 2), probably offers some considerable protection against the uncontrolled deregulation of complement through the alternative pathway amplification loop.

The identified rs12614 and rs641153 CFB variants introduced two different amino acid substitutions for the same residue R32. Both have been studied as sources of protection in complement mediated pathologies such as AMD [52,53,54,55]. The rs12614 (R32W) and rs641153 (R32Q) CFB induced amino acid substitutions weakened the low affinity interaction and contributed to the disruption of the dynamics that contribute to the C3bBb pre-convertase assembly [52].

We believe that among the three study groups examined, the increased frequencies of the rs1061170, rs1065489, rs800292 CFH coding SNPs in the MIS-C group are potentially predisposed to complement deregulation, which results in poorer opsonization without, however, a deregulated alternative pathway amplification loop. In MIS-C individuals, the poorer opsonization of the virus may result in slower and impaired immune clearance with sustained systemic inflammation, however, without any complement diminishment phenomena associated with severe infection [5].

This study is probably one of the first to study and describe complement variants in children with COVID-19 regardless of the severity and clinical manifestations. Based on this, we can possibly distinguish those patients at risk for the onset of serious manifestations and complications of the disease, who could potentially benefit from the administration of complement inhibitors, in line with the studies and efforts made in adults. In conclusion, we detected combinations of coding variants in complement genes in pediatric patients with COVID-19 that have previously been described in inflammatory and endothelial dysfunction syndromes. Although the landscape of COVID-19 infection is constantly evolving with new virus variants and added rounds of vaccinations that influence the clinical outcomes of the disease, we nevertheless present robust findings in a homogeneous Caucasian population that are in accordance and expand the previous knowledge. Although the patterns detected in this pilot study are interesting, the relatively small size of the MIS-C group (*nM* = 7) does not allow for broader generalizations of these early observations and further studies of scale are required for more advanced mechanistic insights.

## Figures and Tables

**Table 1:** A summary of the pediatric COVID-19 patients examined in this study.

Characteristics of the Pediatric COVID-19 Patients Examined in this Study (*nT* = 71)			
Characteristics	Non-Admitted (*N*) *nN* = 32	Hospitalized ^1^ (*Y*) *nY* = 32	Hospitalized + MIS-C Pathology ^2^ (*M*) *nM* = 7
**Age (years),** **(Median range)**	7 (0.3–16)	7 (0.3–17)	4.7 (2–12)
**Sex, Male:female**	17:15	19:13	5:2
**Fever**	28	29	7
**Dyspnea onset**	0	0	6
**Cough**	24	8	6
**White Blood Count (WBC) (×10^3^/μL),** **(Median range)**	7.045 (4.030–11.780)	7.215 (3.900–19.080)	7.900 (3.500–11.600)
**Platelets (×10^3^/μL),** **(Median range)**	258 (100–351)	258 (30–715)	95 (62–155)
**Need for treatment**	2	15	7

1. Admitted, 2. MIS-C: COVID-19 induced Multisystem Inflammatory Syndrome in Children (MIS-C) (CDC, HAN Archive-00432).

**Table 2:** The human alternative pathway (AP) associated complement missense coding SNPs detected in pediatric COVID-19 patients.

Complement Alternative Pathway (AP) Missense Coding Variants Identified in Pediatric COVID-19 Patients								
8 Common AP Variants among the Three Study Groups								
rsID ^1^	Gene ^2^	Variant Frequency-VF% (Repeats/Patients-R/P) ^3^				Encoded Mutation		
		Non-Admitted (*N*) *nN = 32*	Admitted (*Y*) *nY = 32*		MIS-C (*M*) *nM = 7*			
**rs1047286**	***C3***	35 (11/32)	31 (10/32)		29 (2/7)	**R102G (beta chain)**		
**rs2230199**	***C3***	35 (11/32)	47 (15/32)		29 (2/7)	**P314L (beta chain)**		
**rs12614**	***CFB***	35 (11/32)	22 (7/32)		57 (4/7)	**R32W (Ba)**		
**rs641153**	***CFB***	9 (3/32)	13 (4/32)		-	**R32Q (Ba)**		
**rs1061170**	***CFH***	81 (26/32)	81 (26/32)		100 (7/7) *	**Y402H (CCP7)** **(Sulphation)**		
**rs1065489**	***CFH***	25 (8/32)	34 (11/32)		57 (4/7)	**E936D (CCP16)**		
**rs800292**	***CFH***	53 (17/32)	47 (15/32)		57 (4/7)	**V62I (CCP1)**		
**rs11098044**	***CFI***	100 (32/32)	100 (32/32)		100 (7/7)	**T308A** **(HC C-terminal end)**		
**18 Unique AP variants in two of the three study groups**								
**Non-Admitted** **(*N*)** ***nN* = 32**			**Admitted** **(*Y*)** ***nY* = 32**			**MIS-C** **(*M*)** ***nM* = 7**		
**rsID ^1^**	**Gene ^2^**	**VF%** **(R/P) ^3^**	**rsID ^1^**	**Gene ^2^**	**VF%** **(R/P) ^3^**	**rsID ^1^**	**Gene ^2^**	**VF% (R/P) ^3^**
**rs117793540**	***C3***	3 (1/32)	**rs765821415**	***C3***	6 (2/32)	**N/A**		
**rs531259592**	***C3***	3 (1/32)	**rs772034590**	***C3***	3 (1/32)			
**rs146803767**	***CD46***	3 (1/32)	**rs772981625**	***C3***	3 (1/32)			
**rs371106885**	***CD46***	3 (1/32)	**rs4151651**	***CFB***	6 (2/32)			
**rs373182738**	***CD55***	3 (1/32)	**rs149101394**	***CFB***	3 (1/32)			
**rs144812066**	***CFB***	3 (1/32)	**rs2230216**	***CFD***	3 (1/32)			
**rs35274867**	***CFH***	3 (1/32)	**rs758829036**	***CFD***	3 (1/32)			
**rs149474608**	***CFH***	3 (1/32)	**rs515299**	***CFH***	3 (1/32)			
			**rs534399**	***CFH***	3 (1/32)			
			**rs149474608**	***CFH***	3 (1/32)			
			**rs74817407**	***CFI***	3 (1/32)			

^1^ Reference SNP ID, ^2^ NCBI Gene database, ^3^ Detected coding SNP frequency in each population. Corresponds to the number of times that each SNP is represented within each of our two major study group populations. In red, the CFH coding SNPs of interest and their frequencies in the MIS-C group. * *p* = 0.007 for the comparison between the MIS-C and non-MIS-C patients. In blue are the protective CFB coding SNPs of interest and their frequencies in the MIS-C group. CF: complement factor. MIS-C: COVID-19 induced Multisystem Inflammatory Syndrome in Children (MIS-C) (CDC, HAN Archive-00432).

**Table 3:** The human lectin pathway (LP) associated complement missense coding SNPs detected in pediatric COVID-19 patients.

Complement Lectin Pathway (LP) Missense Coding Variants Identified in Pediatric COVID-19 Patients								
9 Common LP Variants among the Three Study Groups								
rsID ^1^	Gene ^2^	Variant Frequency-VF% (Repeats/Patients-R/P) ^3^				Encoded Mutation		
		Non-Admitted (*N*) *nN* = 32		Admitted (*Y*) *nY* = 32	MIS-C (*M*) *nM* = 7			
**rs7567833**	***COLEC11***	9 (3/32)		3 (1/32)	29 (2/7)	**H219R,** **C-type lectin domain**		
**rs532781899**	***FCN3***	13 (4/32)		16 (5/32)	-	**L117S (Del)/** **L117P (Ins),** **Indel**		
**rs72549154**	***MASP1/3***	13 (4/32)		9 (3/32)	-	**R576M,** **MASP-1 Isoform 2→MASP-3**		
**rs12711521**	***MASP2***	84 (27/32)		88 (28/32)	71 (5/7)	**D371Y, Isoform 1:** **364–432: CCP2**		
**rs139962539**	***MASP2***	3 (1/32)		6 (2/32)	-	**T294M, Isoform 1:** **184–296: CUB 2**		
**rs2273346**	***MASP2***	3 (1/32)		6 (2/32)	14 (1/7)	**V377A, Isoform 1:** **364–432: CCP2**		
**rs72550870**	***MASP2***	9 (3/32)		6 (2/32)	-	**D120G, Isoforms 1, 2:** **16–137: CUB 1**		
**rs1800450**	***MBL2***	41 (13/32)		19 (6/32)	43 (3/7)	**G54D, 42–99:** **Collagen-like domain**		
**rs5030737**	***MBL2***	16 (5/32)		22 (7/32)	14 (1/7)	**R52S/R52C, 42–99:** **Collagen-like domain**		
**8 Unique LP variants in two of the three study groups**								
**Non-Admitted** **(*N*)** ***nN* = 32**			**Admitted** **(*Y*)** ***nY* = 32**			**MIS-C** **(*M*)** ***nM* = 7**		
**rsID ^1^**	**Gene ^2^**	**VF%** **(R/P) ^3^**	**rsID ^1^**	**Gene ^2^**	**VF%** **(R/P) ^3^**	**rsID ^1^**	**Gene ^2^**	**VF%** **(R/P) ^3^**
**rs113269917**	***COLEC11***	3 (1/32)	**rs148649884**	***FCN1***	3 (1/32)	**N/A**		
**rs28945068**	***MASP1***	3 (1/32)	**rs758432223**	***MASP1***	3 (1/32)			
**rs147270785**	***MASP2***	6 (2/32)	**rs185903926**	***MASP2***	6 (2/32)			
**rs137974936**	***MBL2***	3 (1/32)	**rs1800451**	***MBL2***	6 (2/32)			

^1^ Reference SNP ID, ^2^ NCBI Gene database, ^3^ Detected coding SNP frequency in each population. Corresponds to the number of times that each SNP is represented within each of our two major study group populations. In green, are the MBL2 rs1800450 coding SNPs of interest and its frequency in the admitted group. COLEC: collectin; FCN: ficolin; MASP: mannose-associated serine protease; MBL: mannose binding lectin; MIS-C: COVID-19 induced Multisystem Inflammatory Syndrome in Children (MIS-C) (CDC, HAN Archive-00432).

## Data Availability

The authors declare that the data supporting the findings of this study are available within the paper and the Supplementary Materials.

## Supplementary Materials

The following supporting information can be downloaded at: https://www.mdpi.com/article/10.3390/cimb44070193/s1.
